# First report on the molecular epidemiology of Malaysian *Staphylococcus epidermidis* isolated from a University Teaching Hospital

**DOI:** 10.1186/1756-0500-7-597

**Published:** 2014-09-03

**Authors:** Nurul Azirah Mohamad Sani, Hassriana Fazilla Sapri, Hui-min Neoh, Salasawati Hussin

**Affiliations:** Department of Medical Microbiology and Immunology, Faculty of Medicine, Universiti Kebangsaan Malaysia Medical Molecular Biology Institute (UMBI), UKM Medical Centre, Bandar Tun Razak, Cheras, Kuala Lumpur, 56000 Malaysia; UKM Medical Molecular Biology Institute (UMBI), Universiti Kebangsaan Malaysia, Kuala Lumpur, Malaysia

**Keywords:** SCC*mec* typing, Virulence gene typing, *Ica* operon characterization, Malaysian *S. epidermidis*

## Abstract

**Background:**

*Staphylococcus epidermidis* is a pathogen associated with nosocomial infections whose medical importance has increased due to progressively invasive medical procedures. In this study, we characterized the molecular epidemiology of *S. epidermidis* strains circulating in our university hospital situated in Kuala Lumpur, Malaysia.

**Findings:**

A total of 798 *S. epidermidis* were isolated from our university hospital, where 56.3% of the isolates were found to be cefoxitin (methicillin) resistant and also positive for the *mecA* gene. Staphylococcus Cassette Chromosome *mec* (SCC*mec*) typing revealed that 39.6% of the methicillin-resistant *S. epidermidis* (MRSE) were SCC*mec*-untypeable, with 54.6% harboring the cassette chromosome recombinase C (*ccrC*) gene. A total of 67 isolates from the neonatal intensive care unit (NICU) was selected for pulsed-field gel electrophoresis (PFGE) typing, where 13 pulsotypes were identified at a cut-off value of 80% similarity. No significant association was found between the PFGE pulsotypes, SCC*mec* types and antibiotic susceptibilities. Polymerase chain reaction (PCR) assays to detect biofilm-associated genes in the *ica* operon and also 4 staphylococcal toxin genes (*cna*, *seh*, PVL genes and *tst-1*) revealed that only 8.0% isolates had the complete operon, while *cna* was the most prevalent toxin gene detected amongst the isolates (35.8%).

**Conclusion:**

To our knowledge, this is the first report on the molecular epidemiology of Malaysian *S. epidermidis*. We found the strains to be low in virulence potential; nevertheless further studies have to be conducted to determine if this phenomenon translates into a better clinical outcome for patients.

## Findings

*Staphylococcus epidermidis*, categorized as coagulase-negative staphylococci (CoNS), are important agents of nosocomial infection as they are human normal flora with abilities to survive in hospital settings and medical devices [1]. Recently, the medical importance of *S. epidermidis* has continued to rise along with the increase of invasive medical procedures and number of immune-compromised patients [2]. In addition, the phenomenon of drug resistance in multi-drug resistant *S. epidermidis* (MDRSE) as well as methicillin-resistant *S. epidermidis* (MRSE) strains, coupled with the ability of the pathogen for biofilm production lead to complications for the treatment and eradication of *S. epidermidis* infections [1, 2]. Molecular studies have also identified the presence of staphylococcal toxin genes, such as exotoxins, enterotoxins and the toxic shock syndrome toxin (*tst-1*) in *S. epidermidis*, elevating the clinical importance of this nosocomial pathogen [3]. As data about the molecular epidemiology of Malaysian CoNS is still lacking and as *S. epidermidis* is the predominant CoNS species for nosocomial infections [1–3], we initiated a study to characterize the molecular epidemiology of *S. epidermidis* strains circulating in our university hospital situated in Kuala Lumpur, Malaysia.

In 2009, a total of 2354 staphylococci infections were recorded in the hospital. The first isolate of each infection (according to the time and date they were received and registered in the hospital diagnostic laboratory) was collected, colony-purified and stocked as strains. From these strains, 1156 (49.1%) were identified as CoNS via the tube coagulase test, where 798 (69.0%) strains were later determined as *S. epidermidis* with PCR using species-specific primers and cycling conditions as described previously [4] . All CoNS infections have been classified as “true” infections and not contaminants by hospital clinical microbiologists via the presence of pus cells in the samples). From medical record review, the *S. epidermidis* infections were found to have occurred in various wards and clinics of the hospital, including eight surgical (12.5%) and six medical wards (25.8%), the adult and neonatal intensive care units (15.0%), four paediatric wards (4.9%) and also eight outpatient clinics (4.2%). The strains were isolated mainly from blood (38.1%), pus (40.7%), tracheal aspirates (3.0%) and tips (2.6%).

The study strains were subsequently tested for their susceptibilities towards various antibiotics using the disk diffusion method (Oxoid Microbiology Products, Thermo Fisher Scientific Inc.) according to Clinical Laboratory Standards Institutes (CLSI) recommendations [5]. More than half (56.3%) of the tested strains were found to be resistant to cefoxitin (1 μg), 81.2% were resistant to penicillin (10 units), while 53.3% were resistant to erythromycin (15 μg). Comparatively, fewer strains were resistant to ciprofloxacin (5 μg) (29.9%), fusidic acid (10 μg) (30.4%), gentamicin (10 μg) (36.0%), mupirocin (5 μg) (24.2%) and rifampicin (5 μg) (12.9%). Interestingly, 1.6% of the strains were resistant to teicoplanin (30 μg); nevertheless, all strains were susceptible to vancomycin (30 μg). Almost half of the strains (48.6%) were multidrug-resistant; the more common drug-resistance combinations were ciprofloxacin-erythromycin-gentamicin-oxacillin resistance (3.9%), ciprofloxacin-erythromycin-fusidic acid-gentamicin-oxacillin resistance (2.5%) and erythromycin-fusidic acid-oxacillin resistance (2.5%).

Genomic DNA of each strain was then extracted for molecular studies. Firstly, we proceeded to type the staphylococcus cassette chromosome *mec (*SCC*mec*) of MRSEs using the protocol from Kondo et al. [6]. All MRSEs were found to harbor the *mecA* gene. However, 39.6% of the MRSE were SCC*mec*-untypeable; this finding is in line with other reports of high untypeable-SCC*mec* percentage among MRSEs (30% - 50%) [1, 7]. A total of 9.4 and 7.6% of the tested strains were successfully typed as SCC*mec* type IV and V, respectively; but only 0.9, 1.6, 1.6, 0.4, 0.9% of the strains were typeable as SCC*mec* types I, II, III, VI and VIII, respectively (Table 1). A total of 33 new potential SCC*mec* combinations were found, and 9 out of these 33 combination patterns have been described elsewhere [7]. Interestingly, even though many of the MRSEs were SCC*mec*-untypeable*,* a high percentage (54.6%) were found to harbor the cassette chromosome recombinase C (*ccrC*) gene, either alone or in combination with the other *ccr* genes, where *ccrA2B2* was the frequent combination partner (43.2%) (Table 2). *ccrC* in SCC*mec* V was reported to have high transferability among *Staphylococcal* species and strains [8]. This edge in transferability might be the reason why most *S. epidermidis* in our hospital harbors the *ccrC*. High abundance of *ccrC* might have also driven the mobility of SCC*mec* elements in our strains, causing the derivement of new and varied SCC*mec* types in the species. In addition, Katayama et al. [9] reported that *ccrA2B2 e*lements usually carry macrolide resistance genes; this tallied with our strains’ antibiogram where 53.3% of our CoNS that carried *ccrA2B2* were also resistant to erythromycin.

**Table 1:** **SCC** ***mec*** **types of methicillin-resistant** ***S. epidermidis*** **used in this study**

SCC***mec***type	Frequency (n)	Percentage (%)
Type I	4	0.9
Type II	7	1.6
Type III	7	1.6
Type IV	42	9.4
Type V	34	7.6
Type VI	2	0.4
Type VIII	4	0.9
Untypeable	178	39.6
New Pattern	171	38.1
Total	449	100

**Table 2:** **New combinations of** ***ccr*** **and** ***mecA*** **class leading to the formation of new SCC** ***mec*** **types in** ***S. epidermidis*** **strains used in this study**

SCC***mec***type	***ccr/mecA***	Percentage, %
New Pattern 1	*ccrA1B1/A*	1.8
New Pattern 2	*ccrA1B1/C*	1.8
New Pattern 3	*ccrA2B2/C*	6.4
New Pattern 4	*ccrA3B3/B*	0.6
New Pattern 5	*ccrA3B3/C*	1.2
New Pattern 7	*ccrA4B4/C*	1.2
New Pattern 8	*ccrC/A*	8.8
New Pattern 9	*ccrC/B*	9.9
New Pattern 10	*ccrA1B1 + ccrA3B3 + ccrC/A*	0.6
New Pattern 11	*ccrA1B1 + ccrA4B4/A*	0.6
New Pattern 12	*ccrA1B1 + ccrA4B4/B*	0.6
New Pattern 13	*ccrA1B1 + ccrA4B4 + ccrC/B*	1.2
New Pattern 15	*ccrA1B1 + ccrA4B4 + ccrC/A*	1.2
New Pattern 16	*ccrA1B1 + ccrC/B*	1.8
New Pattern 17	*ccrA1B1 + ccrC/C*	4.1
New Pattern 19	*ccrA2B2 + ccrA3B3 + ccrC/A*	0.6
New Pattern 20	*ccrA2B2 + ccrA4B4/A*	1.8
New Pattern 21	*ccrA2B2 + ccrA4B4/B*	4.1
New Pattern 22	*ccrA2B2 + ccrA4B4 + ccrC/A*	1.2
New Pattern 23	*ccrA2B2 + ccrA4B4 + ccrC/B*	2.9
New Pattern 24	*ccrA2B2 + ccrA4B4 + ccrC/C*	1.2
New Pattern 25	*ccrA2B2 + ccrC/A*	2.9
New Pattern 26	*ccrA2B2 + ccrC/B*	26.3
New Pattern 27	*ccrA2B2 + ccrC/C*	7.0
New Pattern 29	*ccrA2B2 + CCRA3B3/B*	0.6
New Pattern 30	*ccrA4B4 + ccrC/A*	3.5
New Pattern 31	*ccrA4B4 + ccrC/B*	2.3
New Pattern 32	*ccrA4B4 + ccrC/C*	3.5
New Pattern 33	*ccrA1B1 + ccrA2B2 + ccrA3B3 + ccrC/A*	0.6
	Total	100

**Figure 1:**
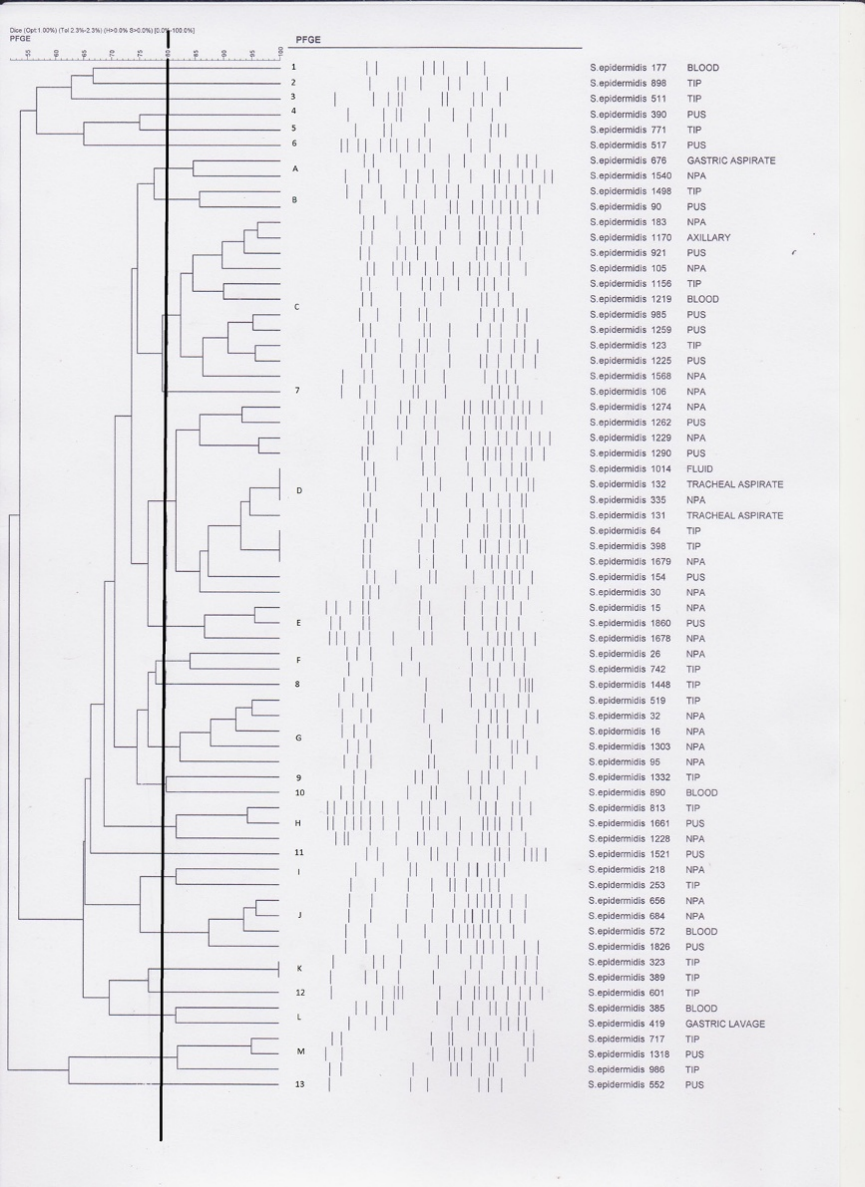
**Pulsed-field gel electrophoresis (PFGE) pulsotypes for 67** ***S.*** ***epidermidis*** **strains isolated from the neonatal intensive care unit (NICU).** With a 80% similarity cut-off point, a total of 13 pulsotypes and 13 singletons were identified. Two major pulsotypes, designated as pulsotypes C and D, contained 15 and 11 strains, respectively.

As no dominant SCC*mec* type could be found for our study strains, we attempted to type the strains using Pulsed-Field Gel Electrophoresis (PFGE) to check for a dominant pulsotype in our hospital setting. Due to the large number of *S. epidermidis* strains available in 2009, only 67 strains from the neonatal intensive care unit (NICU) were selected for PFGE typing, as most of the strains used in this study were isolated from this ward. PFGE was carried out using a protocol as described previously [1]. When the isolates were analyzed at 80% similarity cut-off point, a total of 13 pulsotypes and 13 singletons were identified, with the 2 major pulsotypes, C and D, containing 15 and 11 strains, respectively (Figure 1). No significant association was found between the strains’ PFGE pulsotypes, SCC*mec* types and antibiotic susceptibilities.

Lastly, to better understand the virulence potential of our study strains, we characterized the biofilm-associated *ica* operon and also determined the presence of 4 staphylococcal toxin genes (*cna*, *seh*, PVL genes and *tst-1*) in these strains, using currently available protocols [2, 10]. Interestingly, only 7.4% of the study isolates did not harbor the *ica* operon, while 8.0% of the isolates had the complete operon. *icaB* was the commonly detected *ica* gene, either singly (12.2%) or in varied combinations with the other *ica* genes (40.0%). Besides *icaA* and *icaD*, *icaB* is also important for proper biofilm formation, as it codes for a deacetylase which maintains the adhesiveness between bacterial cells and biofilm [2]. On the other hand, we also noted a high abundance (85.1%) of the insertion sequence IS*256* in this operon of our isolates, a phenomenon which was more frequently observed in nosocomial *S. epidermidis* strains compared to commensal strains [11]. As for staphylococcal toxin gene determination, 62.7% of the strains did not carry any of the 4 virulence genes determined, while *cna* (35.8%) was the most prevalent toxin gene detected in the strains, either singly (19.4%) or in combination with the other toxin genes of *tst-1*, *seh* and PVL genes (*cna* with *tst-1*, 1.5%; *cna* with *seh*, 7.5%; *cna* with PVL genes, 4.5%; *cna* with *seh* and *tst-1*, 3.0%).

To our knowledge, this is the first report about the molecular epidemiology of Malaysian *S. epidermidis* isolated from a university teaching hospital. The *S. epidermidis* studied in our investigation had diverse SCC*mec* elements and were somewhat low in virulence potential in terms of low staphylococcal virulence gene carriage and haboring incomplete *ica* operons. Nevertheless, it remains to be determined if these findings do translate into a better clinical outcome for the infected patients.
